# The dental phenotype of primary dentition in SATB2-associated syndrome: a report of three cases and literature review

**DOI:** 10.1186/s12903-022-02594-4

**Published:** 2022-11-22

**Authors:** Xiaojing Li, Xiaowei Ye, Jimei Su

**Affiliations:** Department of Stomatology, The Children’s Hospital, Zhejiang University School of Medicine, National Clinical Research Center for Child Health, Hangzhou, 310052 Zhejiang China

**Keywords:** SATB2-associated syndrome, *SATB2* gene, Dental abnormalities

## Abstract

**Background:**

SATB2-associated syndrome (SAS; OMIM: 612,313) is an autosomal dominant inherited multisystemic disorder caused by several variants of the *SATB2* gene. SAS is characterized by intellectual disability, developmental delay, severe speech anomalies, craniofacial anomalies, and dental abnormalities. Here, we report the dental phenotype of primary dentition of three Chinese children with SAS.

**Case presentation:**

All three cases with SAS showed intellectual disability, speech and language anomalies, and palate anomalies. For the dental phenotype, all three cases showed macrodontia, crowded dentition, extensive caries, periapical abscesses and fistulas. Radiographs showed the wide-open root apex of deciduous teeth, loss of mandibular second bicuspids, delayed root formation of permanent teeth, rotated teeth, and taurodontism. Sanger sequencing of case 1 showed that there was a heterozygous code shift variation, c1985delT (p.F662Sfs*9) in the *SATB2* gene, which has not been reported in literature. Root canal therapy, carious restoration, and teeth extraction were managed promptly, while preventive dental care was given regularly.

**Conclusions:**

The dental phenotype of primary dentition in SAS may show macrodontia, crowded dentition, severe caries, wide-open root apex of deciduous teeth, loss of mandibular second bicuspids, delayed root formation of permanent teeth, rotated teeth, and taurodontism. Regular oral hygiene instructions and preventive dental care are both required.

## Background

Special AT-rich sequence binding 2-associated syndrome (SATB2-associated syndrome, SAS, OMIM: 612,313) is a multisystemic disorder, inherited by an autosomal dominant pattern caused by several variants of the *SATB2* gene and at the chromosome 2q33.1 [1]. This syndrome was first described in a 16-year-old male with an interstitial deletion of 2q32.2-2q33.1 and severe intellectual disability by Glass, so the syndrome can also be described as Glass syndrome [2]. Its most common features include intellectual disability, developmental delay, severe speech anomalies, craniofacial anomalies, and dental abnormalities [3]. These individuals may also suffer from behavioral problems, feeding difficulties, epilepsy, skeletal anomalies, growth restriction, and congenital heart defects [4–6].

For palatal and dental abnormalities, clinical manifestations include cleft palate, high-arched palate, macrodontia, delayed tooth eruption, malformed teeth, missing teeth, sialorrhea, and bruxism [7, 8]. However, there are few reports about the dental phenotype of primary dentition. Here, we present three cases of SAS with primary dentition.

## Case presentation

The clinical features of three described cases with SAS are shown in Table 1.

**Table 1:** Clinical features of described patients with SAS

Patient	Case 1	Case 2	Case 3
Gender	F	F	M
Age at diagnosis, years	2.5	2	1
Current age, years	4	6	7
Nucleotide	c1985delT	c1196G > A	c1286G > A
Protein	F662Sfs*9	Arg399His	Arg429Gln
Inheritance	De novo	De novo	De novo
Variant reference	This report	Zarate et al	Zarate et al
DD/ID	+	+	+
Speech development disorder	+	+	+
Behavior anomalies	+	−	−
Sleep difficulties	−	−	−
Abnormalities on Brain imaging	Cyst of pellucid septal cave	Cyst of pellucid septal cave	−
Abnormal EEG	−	−	−
Growth retardation	+	+	−
Seizures	−	−	−
Micrognathia	−	−	−
Cleft palate	−	+	+
High-arched palate	+	−	−
Macrodontia	+	+	+
Crowding teeth	+	+	+
Missing teeth	+	+	−
Skeletal abnormalities	−	−	−

*DD* Developmental delay, *ID* Intellectual disability, *ND* Not done, *EEG* Electroencephalogram

### Case 1

A 4-year-old female was referred to our department with periapical abscesses of anterior teeth for one month. The patient was born at full term by cesarean section, with a weight of 3.2 kg and a height of 49 cm, who was able to walk at approximately 2-year-old. The female was diagnosed with intellectual disability, language development disorder when she was 2 years and 7 months old, and then received 6 months of rehabilitation training in our hospital. Sanger sequencing showed that the patient’s *SATB2* gene had a code shift variation, c1985delT (p.F662Sfs*9) and was diagnosed with SAS. However, the mutation was not present in the patient’s parents.

**Fig. 1:**
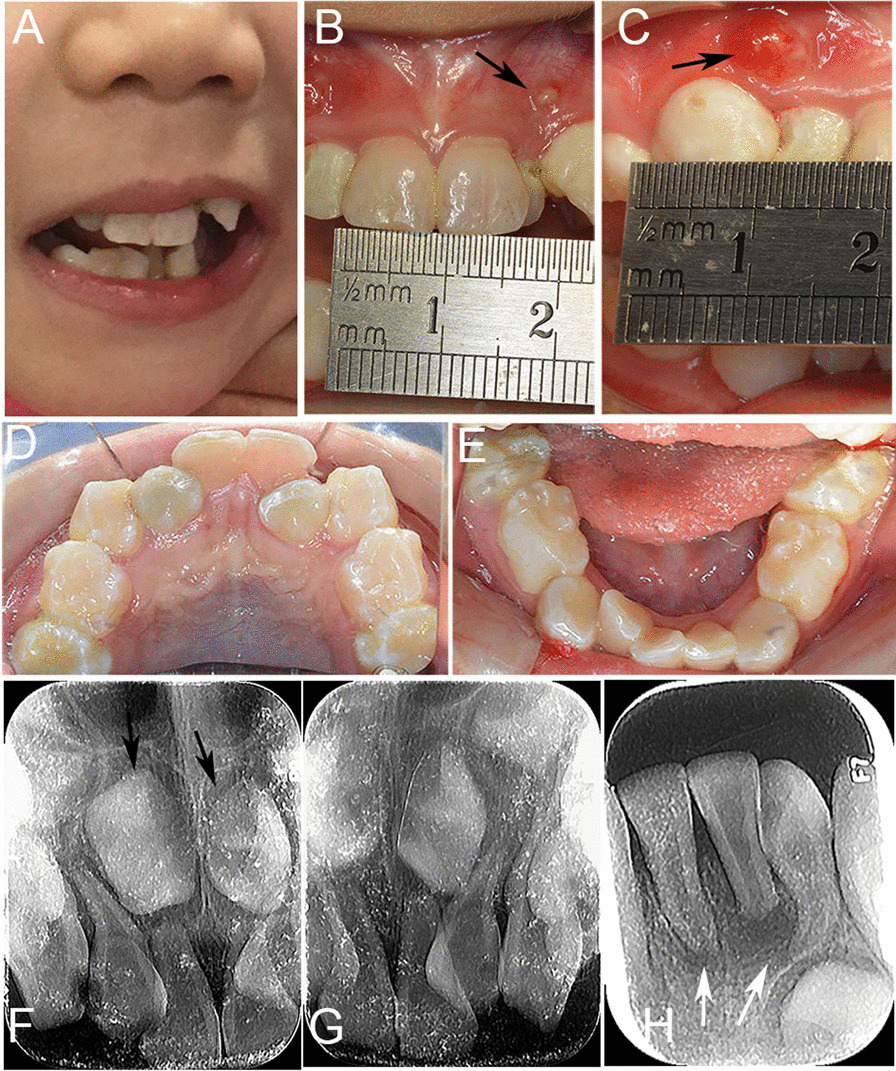
Clinical and radiographic features of case 1 with SAS. **A** The extraoral photo showing a flat philtrum, and a thin upper lip; **B**–**E** The intraoral photos showing macrodontia, crowded teeth, caries and periapical abscesses (black arrows); **F**–**H** The periapical radiographs of deciduous anterior teeth showing wide-open root apex and periapical low-density image (white arrows), and malformed and inverted permanent teeth 11 and 21 (black arrows), with the missing tooth buds of permanent teeth 31 and 41

Physical examination of this 4-year-old female showed a height of 99 cm and a weight of 15 kg, along with the presence of mental retardation, and language development disorder. The extraoral examination revealed a flattened philtrum with a thin upper lip (Fig. 1A). The intraoral examination revealed a high-arched palate, malocclusion, crowded teeth, missing deciduous tooth 82. Her teeth were larger teeth than normal, with proximal and distal diameters of 8 mm for the right upper deciduous central incisor, 10 mm for the right upper deciduous canine (Fig. 1B–E). In addition, the patient had poor oral hygiene, and deciduous teeth 51, 52, 53, 61, 62, 63 and 74 were decayed. Periapical abscesses were observed on displaced maxillary deciduous teeth 52 and 62.

The periapical radiograph of the maxillary anterior teeth showed low apical density image of deciduous teeth 52 and 62, wide-open root apex of deciduous teeth 51, 52, 61, 62, and malformed and inverted tooth buds of permanent teeth 11 and 21 (Fig. 1F–G). Similar features were observed in mandibular anterior teeth, including a periapical low-density image and wide-open root apex, and the root of tooth 72 was dilacerated (Fig. 0.1H). Nevertheless, tooth buds were not observed in permanent teeth 31 and 41.Taken together, the patient was diagnosed with SAS, chronic periapical periodontitis and caries. The treatment consisted of extraction of tooth 62, root canal therapy of tooth 52, and restoration of the other caries. After three months of treatment, periapical abscesses of the patient’s deciduous teeth 52 and 62 were healed.

### Case 2

A 6-year-old female with severe caries and malposed teeth was referred to our department. The patient was born at full term by normal vaginal delivery and was 49 cm tall, weighing 3 kg. The patient was able to walk at 17 months, and was found to have growth retardation, mental retardation, and limited speech and language development. She was subsequently diagnosed with SAS and underwent cleft palate surgery. A missense variation of the *SATB2* gene c1196G > A (p.Arg399His) was detected in the patient by sanger sequencing. However, the same variation and similar features were not present in the patient’s parents.

**Fig. 2:**
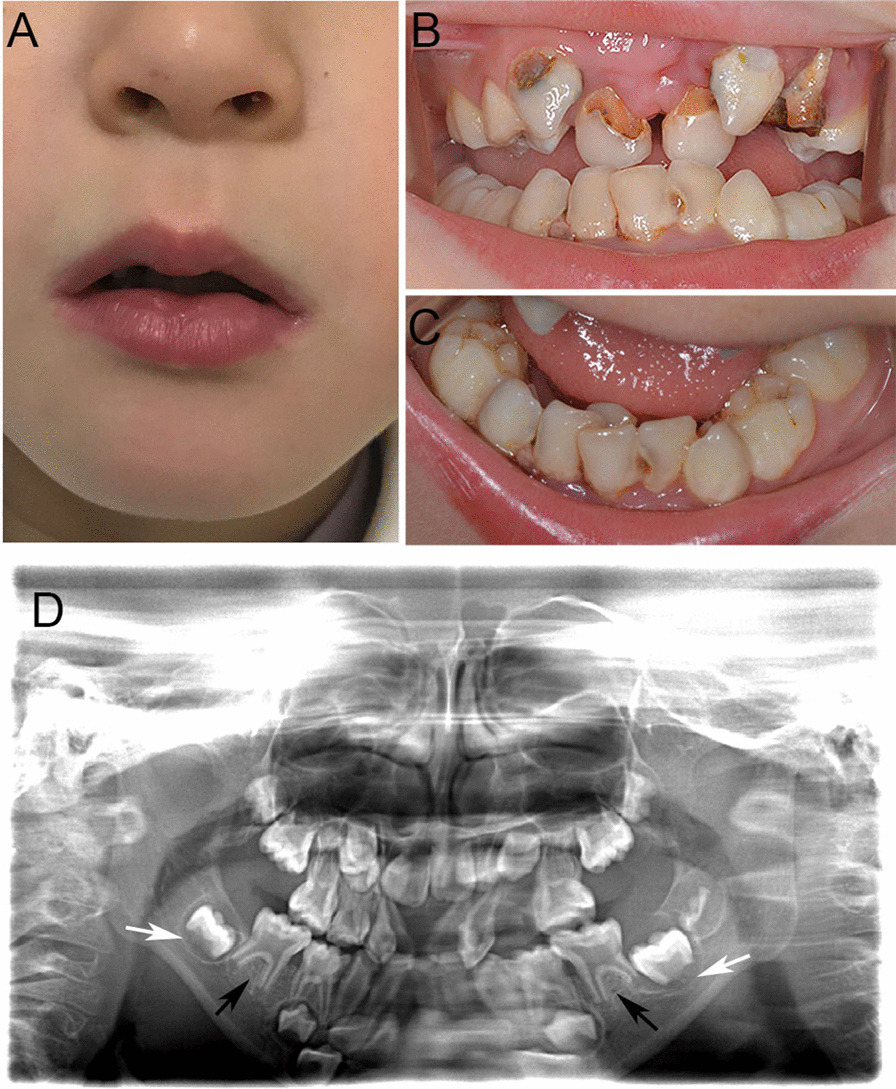
Clinical and radiographic features of case 2 with SAS. **A** The extraoral photo showing cherry-like lips and thin upper lip; **B**–**C** The intraoral photo showing extensive caries, crowed teeth, malposed teeth and poor oral hygiene; **D** Panoramic radiographs showing loss of mandibular second bicuspids, delayed root formation of primary teeth 75 and 85 (black arrows), and permanent teeth 36 and 46 (white arrows)

The patient was 105 cm tall and weighed 16 kg when she came to our department. Physical examination revealed a cherry-like lip and thin upper lip (Fig. 2A). Extensive caries including 52, 53, 54, 62, 63, 64, 71, 72, 73, 74, 75, 81, 82, 83, 84, 85, crowded teeth, and poor oral hygiene were observed in the oral cavity (Fig. 2B–C). Teeth 51 and 61 were missing, and teeth 52 and 62 were dislocated palatally. Panoramic radiographs showed loss of mandibular second bicuspids, delayed root formation of primary and permanent teeth, and taurodontism (Fig. 2D). The patient was diagnosed with SAS, chronic periapical periodontitis and dental caries. Treatment included extraction of teeth 52, 62, 64 under local anesthesia and restoration of other caries. The patient was advised to improve her oral hygiene with regular dental care and fluoridation treatment.

### Case 3

A 7-year-old male with a chief complaint of severe dental caries was referred to our department. The patient was born at full term in normal vaginal delivery, with a height of 50 cm and a weight of 3 kg. He was able to raise his head at 6 months, sit at 7 months, crawl at 2 years old, and walk at 3 years old. His first deciduous tooth erupted at 16 months. He was later found to have growth retardation, mental retardation, and speech and language developmental disorder, and was diagnosed with SAS. The male underwent cleft palate surgery when he was 10 months old, and rehabilitation training for gross motor and language development has continued to the present. Sanger sequencing showed that the patient had a missense variation of SATB2, c1286G > A (p.Arg429Gln). Similar mutations or features were not detected in the patient’s patents.

**Fig. 3:**
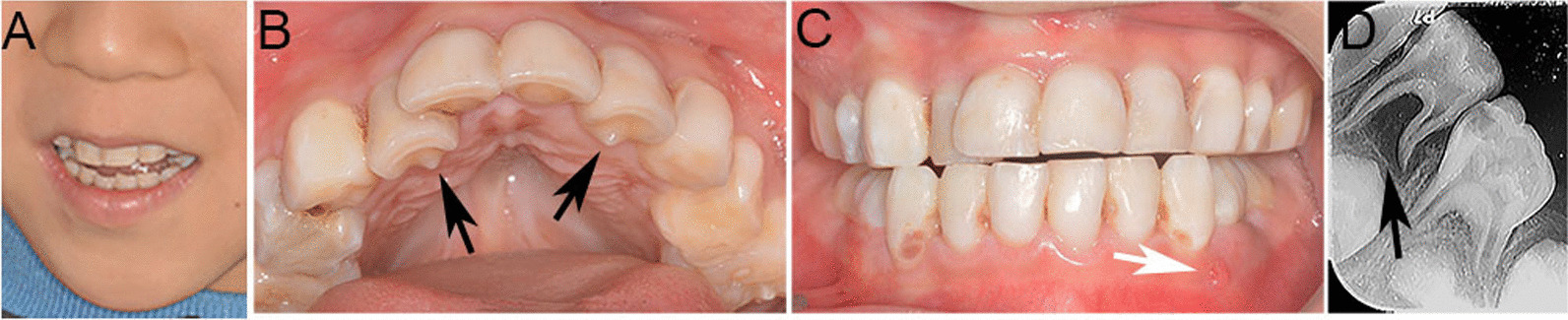
Clinical and radiographic features of case 3 with SAS. **A** The extraoral photo showing a long face, long philtrum, and a thin upper lip; **B–C** The intraoral photo showing large teeth, crowded teeth, talon cusp of teeth 52 and 62 (black arrows), proximal caries, and fistulas and draining abscesses (white arrow); **D** Periapical radiographs showing the large canal and pulp cavity of tooth 74, low-density image in root apical of deciduous tooth 74, wide-open root apex of deciduous molar tooth 75

The patient was 120 cm tall and weighed 21 kg when he came to our department. The extraoral examination revealed a long face, a long philtrum, and a thin upper lip (Fig. 3A). Large teeth, crowded teeth, talon cusps of teeth 52 and 62, proximal caries on teeth 51, 52, 53, 54, 61, 62, 63, 64, 71, 72, 73, 81, 82, 83, and fistulas and draining abscesses in the mandibular deciduous canine region were detected by the intraoral examination (Fig. 3B–C). Periapical radiographs showed the large root canal and pulp cavity of tooth 74, the low density image in the apex of deciduous tooth 74, and the wide-open root apex of deciduous molar tooth 75 (Fig. 3D). Collectively, the patient was diagnosed with SAS, chronic periapical periodontitis and caries. The tooth 74 was extracted and space was maintained, while other carious teeth were restored.

## Discussion and conclusions

The *special AT-rich sequence-binding protein 2* (*SATB2*) gene is located on chromosome 2q33.1, which encodes an evolutionarily highly conserved protein of 733 amino acids with two CUT domains and one homeodomain [9]. The SATB2 protein is a DNA-binding protein, animal models show that it is highly expressed in the brain, the developing upper and lower jaw including the palatal and dental buds, as well as in sites of bone formation and in various tissues including kidney and gut [10–12]. Previous studies have shown that SATB2 protein is also expressed in odontoblasts, predentin, dental pulp cells and blood vessels of human teeth, and plays an important role in odontoblast differentiation and dentin mineralization during tooth development [13]. Thus, in 2017, Zarate summarized the main features with the acronym S.A.T.B.2: S, severe speech anomalies; A, Abnormalities of the palate; T, teeth anomalies; B, behavioral issues with or without Bone or Brain MRI anomalies, and age of onset before age 2.

Pathogenic variants in the *SATB2* gene are numerous, including single nucleotide variants, missense variants, intragenic deletions, translocations, frameshift mutations, splice site alterations, intergenic duplications, and single in-frame alterations [14]. Here, we report three cases with 1 frameshift mutation (case 1) and 2 missense variants (case 2 and case 3) in the *SATB2* gene. These two missense variants, c1196G > A (p.Arg399His) and c1286G > A (p.Arg429Gln), within the first CUT domain of the *SATB2* gene, which is critical for recognizing and binding DNA to regulate gene expression, and these mutations have been reported in the literatures. However, the frameshift mutation, c1985delT (p.F662Sfs*9) of case 1 within the homeodomain of the *SATB2* gene has not been studied in published online databases including ClinVar database, and Human Gene Mutation. Therefore, we consider c1985delT in the *SATB2* gene to be a novel mutation that expands the SATB2 mutation spectrum. Interestingly, we did not detect any mutations in the parents of the three patients, which led us to speculate that the genetic mutations in these three patients were caused by de novo mutations.

A large body of studies have investigated the correlation of genotype and phenotype in the SAS, but no formal correlation has been established [15, 16]. Regardless of genotypes, consistent phenotypes in patients with SAS include intellectual disability(ID)/developmental delay(DD), severely limited speech, palatal abnormalities, and teeth anomalies [15]. For individuals with missense variants, early genotype–phenotype correlations in the first CUT domain of the *SATB2* gene showed a lower frequency of cleft palate [16], however, both case 2 and case 3 with missense variants in this report showed cleft palate, which is inconsistent with previous research. The thin vermilion of upper lip, flat philtrum, prominent chin, deeply set eyes, and micrognathia are the most common facial phenotypes in patients with SAS [15]. The three cases in this report have thin upper lips, and flat and long philtrum, which are in agreement with the previous literatures.

For the dental phenotypes of SAS patients, regardless of genotype, the most consistent features include macrodontia, crowded dentition, delayed tooth eruption, and delayed root formation of the permanent tooth [7, 8]. This report presents dental phenotypes including malocclusion, crowded dentition, larger-than-normal teeth, malposed teeth, talon cusp, extensive caries and poor oral hygiene. Radiographic features showed delayed root formation in deciduous and permanent teeth, large pulp chambers and canals, wide-open root apex of deciduous teeth, malformed and rotated permanent teeth, taurodontism, loss of lower anterior teeth, loss of mandibular second bicuspids. These manifestations are almost in line with many previous reports. These dental features are not found to vary by genotype, and the correlation needs to be validated with more reports. Teeth crowding is mostly thought to be related to macrodontia, and part of it is related to craniofacial growth and development, which needs to be further confirmed. Nevertheless, all 3 cases have extensive caries, while case 1 and case 3 have fistulas and draining abscesses, partly due to poor oral hygiene in these patients, which resulted in poor dental care. In addition, malformed crowns, large pulp chambers and canals, wide-open root apex of deciduous teeth, and delayed root development may increase prevalence of caries and periapical periodontitis.

For treatment, a multidisciplinary approach is essential to treat the developmental delays, speech and language developmental disorder, craniofacial anomalies, dental abnormalities and other medical histories. Oral hygiene instruction and prevention programs should be implemented on a regular basis.

In conclusion, the dental phenotype of primary dentition in SAS may be manifested as macrodontia, crowded dentition, severe caries, wide-open root apex of deciduous teeth, loss of mandibular second bicuspids, delayed root formation of permanent teeth, rotated teeth, and taurodontism. Regular oral hygiene instruction and preventive dental care are required.

## Data Availability

All data generated or analyzed during this study are included within the manuscript.

## Abbreviations

SATB2: Special AT-rich sequence-binding protein 2
SAS: SATB2-associated syndrome
